# Antisense and Functional Nucleic Acids in Rational Drug Development

**DOI:** 10.3390/antibiotics13030221

**Published:** 2024-02-27

**Authors:** Robert Penchovsky, Antoniya V. Georgieva, Vanya Dyakova, Martina Traykovska, Nikolet Pavlova

**Affiliations:** Laboratory of Synthetic Biology and Bioinformatics, Faculty of Biology, Sofia University, “St. Kliment Ohridski”, 8 Dragan Tzankov Blvd., 1164 Sofia, Bulgaria

**Keywords:** antisense oligonucleotides, antisense therapies, drug delivery, drug discovery, nucleic acid engineering, ribozymes, riboswitches

## Abstract

This review is focused on antisense and functional nucleic acid used for completely rational drug design and drug target assessment, aiming to reduce the time and money spent and increase the successful rate of drug development. Nucleic acids have unique properties that play two essential roles in drug development as drug targets and as drugs. Drug targets can be messenger, ribosomal, non-coding RNAs, ribozymes, riboswitches, and other RNAs. Furthermore, various antisense and functional nucleic acids can be valuable tools in drug discovery. Many mechanisms for RNA-based control of gene expression in both pro-and-eukaryotes and engineering approaches open new avenues for drug discovery with a critical role. This review discusses the design principles, applications, and prospects of antisense and functional nucleic acids in drug delivery and design. Such nucleic acids include antisense oligonucleotides, synthetic ribozymes, and siRNAs, which can be employed for rational antibacterial drug development that can be very efficient. An important feature of antisense and functional nucleic acids is the possibility of using rational design methods for drug development. This review aims to popularize these novel approaches to benefit the drug industry and patients.

## 1. Introduction

Nucleic acids have been suitable tools for engineering biosensors for various in vitro and in vivo applications over the last two decades. At the same time, new natural mechanisms for the control of gene expression have been discovered based on various types of RNAs, including micro(mi)RNAs, small interfering(si)RNAs, riboswitches [1], and ribozymes. There are currently four different nucleic acid engineering strategies to inhibit the expression of specific RNAs in the cell, such as siRNAs [2], antisense oligonucleotides (ASOs) [3], ribozymes [4,5], and CRISPR-Cas9 systems [6]. All tools used for nucleic acid-based drug development are inherited based on rational design methods, being novel and the main point of this review.

This review discusses all distinct strategies for RNA inhibition and the engineering methods that make them possible. We also describe the applications of the mRNA inhibition approaches in drug development [7,8], providing validated, proven examples.

Nucleic acids have unique properties that play two essential roles in drug discovery, including drug targets and drugs. Various RNAs can be employed as drug targets, including messenger, ribosomal, non-coding RNAs, ribozymes, riboswitches, etc. Various antisense and functional nucleic acids can be employed as RNA targeting tools. Thus, nucleic acids can be drug targets and drugs themselves.

RNA has the most diverse roles of all biomacromolecules in the cell. The most important roles of RNA are the transcription and translation of genetic instructions involving messenger (m), transfer (t), and ribosomal (r) RNAs. RNAs can also have a catalytic function through the ribozymes and gene regulatory roles through the non-coding (nc) RNAs and the riboswitches. The new high-throughput sequencing and many bioinformatics and biochemical methods are new insights into the role of RNA in biological systems [9].

RNA can exhibit a catalytic function when it works as a ribozyme and a biosensing function as a riboswitch. Naturally occurring RNAs, such as the glmS gene control element, have biosensing and catalytic functions [10]. The proteins are usually responsible for the catalytic and biosensing functions in the cell.

RNA executes essential and complex biochemical functions in the cell and uniquely combines different biochemical properties. Functional RNAs like riboswitches can form complex tertiary structures similar to proteins. For instance, riboswitches can precisely sense the presence of small molecules in the cell, such as guanine, adenine, thiamine pyrophosphate (TPP), and many others, as proteins can. RNA can also specifically hybridize with other nucleic acids [10]. Thus, some RNAs work via Watson-Crick base-paring while others operate via their 3D structures. These unique biochemical properties make functional RNA molecules very promising targets for drug discovery.

RNA is less chemically stable than DNA due to a 2′-hydroxyl group of the ribose, which, in inline conformation, leads to transesterification of the phosphodiester bond of RNA [11]. Many catalytic RNAs, such as hammerhead ribozymes, can speed up the transesterification reaction up to a million times [11].

The mRNA destabilization is an essential mechanism in regulating gene expression in eukaryotic and prokaryotic organisms. However, for eukaryotic organisms that possess RNA-interfering pathways for sequence-specific mRNA decay [12], the non-coding RNAs (ncRNAs) are classified into three groups such as long non-coding RNA (lncRNA), short non-coding RNA (sncRNA), and translational/structural RNA, which possess diverse functions [13]. Their regulation can enhance treatments against different diseases by chromatin modification, inducing immunity via RNA-based vaccination, targeting mRNA for its cleavage via antisense oligonucleotides (ASOs), RNA alternative splicing, RNA masking, shRNA or miRNA-based gene silencing, and transcriptional or translational interference [14,15]. The discovery of gene silencing by ncRNAs in many different organisms, from plants to mammalians, including humans, has extended our understanding of the role of RNA in the cell and given us the opportunities to use non-coding RNA molecules as new targets for drug discovery and drug development.

Understanding RNA interference (RNAi) mechanisms for controlling gene expression has provided us with novel molecular tools that can be used to develop novel drugs. The RNAi pathway regulates mRNA stability and translation in human cells. siRNAs could trigger the RNA silencing of specific genes [16].

Rational design approaches of functional and ASOs can significantly reduce the time and overall cost and increase the rate of successful drug development.

## 2. ASO-Based Strategies for Drug Development

**Figure 1:**
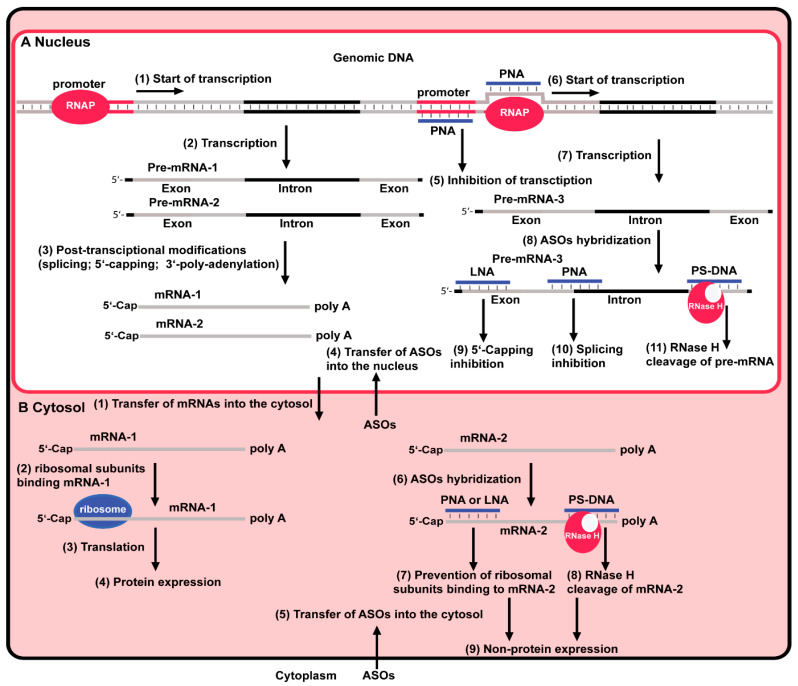
Control of gene expression in eukaryotes by ASOs. (**A**) In the nucleus, genomic DNA has promoter sites. The binding of RNAP to the promoter site (1) triggers pre-mRNA-1 and pre-mRNA-2 (2). These pre-mRNAs undergo post-transcriptional modifications and become mRNA-1 and mRNA-2 (3). ASOs can be transferred into the nucleus (4). One of these ASOs, PNA, can bind to the promoter and inhibit transcription (5). PNA can bind to one of the DNA strands (6) and induce the transcription of pre-mRNA-3 (7). ASOs present in the nucleus can hybridize to complementary sequences of pre-mNA-3 (8); hybridizing of LNA leads to 5‘-capping inhibition (9); hybridizing of PNA leads to splicing inhibition of mRNA-3 (10); hybridizing of PS-DNA leads to the formation of a chimeric structure that is a substrate for RNase H, which recognizes, binds and cleaves mRNA-3 (11). (**B**) The spliced mRNAs are transferred from the nucleus (1). Ribosomal subunits recognize RBS and bind (2). The ribozyme binding triggers translation (3) and protein expression (4). When ASOs are transferred from the cytoplasm into the cytosol (5), they hybridize with complementary sequences of mRNA-2 (6); hybridizing of LNA leads to the prevention of ribosomal binding to mRNA-2 (7); hybridizing of PS-DNA leads to the formation of a chimeric structure (duplex) which is a substrate for RNase H, which recognizes, binds and cleaves mRNA-2 (8) leading to non-protein expression (9).

mRNAs are universally present in all forms of life. Therefore, they are more often becoming targets for treating various RNA-associated diseases such as cancer, neurodegenerative diseases, and many others. One promising method that has attracted more attention in the past decade is antisense oligonucleotide technology (ASOT), which has various applications in drug development, including specific gene silencing [17]. ASOs are single-stranded synthetic oligonucleotides that hybridize to a particular mRNA or other type of RNA and inhibit gene expression [18]. Typically, the length of an ASO is between 15 and 25 nucleotides, which, via Watson-Crick base-pairing, binds complementarily to the target RNA to form a duplex. The ASO-based inhibition is achieved by steric blocking of translation or splicing or by cleavage of the target RNA in the duplex via the RNase H or RNase P enzymes, exon skipping [19], and exon inclusion (Figure 1). Apart from that, ASO can be employed to block the transcription by targeting the genome DNA (Figure 1A).

The unmodified *ASOs* quickly degrade due to the circulating nucleases and are rapidly excreted by the kidneys. This makes them unsuitable for creating therapeutic drugs. Hence, chemical modifications play a crucial role in increasing the stability of ASOs in vivo and the success of an antisense strategy [20]. According to their modifications, there are three generations of antisense oligonucleotides.

### 2.1. First-Generation ASOs

**Figure 2:**
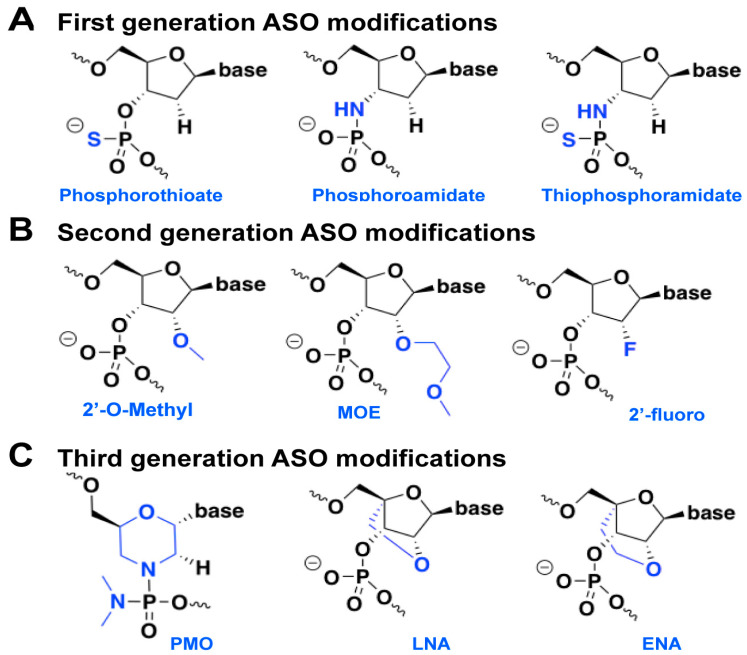
Chemical modifications of various ASOs. (**A**) First-generation ASOs: phosphorothioate, phoshoroamidate, and thiophosphoramidate. They contain phosphodiester bonds in which one of the non-bridging oxygen is replaced by a sulfur atom, an amide group, and a sulfur and amide group. (**B**) In the second-generation ASOs, the nuclease resistance is increased by 2′-alkyl modifications of the ribose and fluorine. (**C**) Third generation ASOs: phosphoramidite morpholino oligomer (PMO), locked nucleic acid (LNA), and ethylene-bridged nucleic acid (ENA).

The first-generation ASO (Figure 2A) has a sulfur atom instead of one of the non-bridging oxygen atoms in the phosphodiester bond (i.e., PS modification). Another substitute for the same oxygen atom can be methyl or amine. This modification makes the ASO more resistant to nucleases and prolongs its half-life in vivo compared with non-modified DNA. The PS-modified ASOs activate RNase H, and, as a result, the target RNA is cleaved right after the hybridization with the ASO under multiple-turnover conditions. However, this generation of ASOs has a few shortcomings, such as the possibility of non-specific binding with proteins by forming S-S bonds and slightly reduced affinity to the target RNAs. The first-generation ASOs have a reduced affinity for mRNA hybridization by approximately 0.5 °C per nucleotide. First-generation ASOs are still widely used in vitro and in vivo, particularly in combination with second-generation ASOs. This combination decreases the shortcomings of first-generation ASOs by reducing the number of PS-modified bases.

### 2.2. Second-Generation ASOs

The second-generation ASO is characterized mainly by 2′-*O*-methyl and 2′-*O*-methoxyethyl (i.e., 2′-alkyl) modifications of the ribose (Figure 2B). These modifications correct the flaws of the previous generation by increasing the specific binding affinity and the hybridization stability of the formed duplex. However, on the other hand, a new disadvantage occurs since the 2′-alkyl modification prevents the cleavage of the target RNA because RNase H cannot be activated and, thus, works under single-turnover conditions. Thus, a chimeric design that combines the first and second generation of modifications is often employed. The ASO has a central part built of a PS-modified 2′-deoxynucleotide and flanking regions on both sides made of 2′-alkyl-modified nucleotides. The main feature is typically between 8 and 16 nt long [21], whereas the flanking wings are between 5 and 10 nt long. Such chimeric ASOs activate RNase H-mediated cleavage while the flanking wings had better protect the ASO from nuclease degradation [22].

### 2.3. Third-Generation ASOs

Third-generation ASO modifications (Figure 2C) mainly involve the furanose ring of the nucleotides. They are primarily electric neutral, unlike the previous two generations, and therefore, they can easily pass the phospholipid bilayer of the cells [23]. Moreover, third-generation ASOs have improved nuclease resistance, target affinity, and pharmacokinetics [24]. Frequently used ASOs from this generation are locked nucleic acid (LNA), peptide nucleic acid (PNA), 2′-*O*,4′-C-ethylene-bridged nucleic acid (ENA), and phosphorodiamidate morpholino oligomer (PMO) (Figure 2C). However, these ASOs cannot induce an RNA cleavage via RNase H and inhibit RNA by blocking translation under single-turnover conditions [22,25].

#### 2.3.1. LNA

LNA follows the Watson–Crick base-pairing rules and forms duplexes with complementary DNA or RNA with increased stabilities and selectivities [26]. Imanishi’s collective presents bicyclic nucleoside analogs—2′-*O*,4′-C-methylene uridine, and -cytidine, with fixed N-type conformation incorporated into oligonucleotides. Typically, 2′-*O*,4′-C-methylene uridine, and -cytidine analogs have a C3′-*endo* sugar puckering, synthesized from uridine [27]. LNA has a modified ribose with an extra bridge connecting the 2′-oxygen and 4′-carbon (Figure 2C). As a result, the ribose is locked in the 3′-end (north) conformation, often found in A-form duplexes, which significantly increases the melting temperature and specificity of hybridization. For instance, LNA/DNA hybrids possess increased T_m_ from 2 °C to 6 °C per monomer compared with DNA/DNA hybrids with the same sequence. LNA/RNA hybrids have even higher T_m_, rising from 3 °C to 8 °C per monomer. LNA oligomers can be synthesized by conventional phosphoramide chemistry, allowing automated synthesis that will be inexpensive when its intellectual property rights expire. Furthermore, LNA oligomers can be easily synthesized as chimeras having DNA, RNA, and other modified bases or labels. For example, two sets of iso-LNA-modified gapmers were tested in HeLa cells for target knockdown activity, systematically changing the number and positions of the long nucleic acid modifications. Based on the structure specifications, the results showed 768 different gapmers targeting the HeLa cells for target knockdown activity and cytotoxic potential because of the binding affinity between the ASOs and the mRNA target [28]. When the accessible regions in the specific target are chosen or identified, it is possible to optimize the LNA gapmers with improved pharmacological profiles to target them.

Usually, the chimeric ASOs, like phosphorothioate DNA, are flanked by stability-enhancing modified nucleotides such as D-2-O-methylribose modifications [29]. Nucleotide modifications of D-2-*O*-methylribose in the central DNA gap could disturb the RNase H function [29]. LNA gapmers can activate RNase H. One of the first experiments conducted with LNA/DNA gapmers was performed on the central nervous system of rats, targeting their delta-opioid receptor mRNA [28]. It was designed superior to dose-dependent and sequence-specific inhibition by an iso-sequential DNA antisense oligonucleotide [30]. Subsequent experiments based on receptor binding showed that LNA, DNA, and PS ASOs reduce the delta-opioid receptor density by 35% to 55%. In another experiment, LNAs/DNAs were tested targeting the 3′-UTR of intercellular adhesion molecule-1 mRNA [31]. Results showed that an LNA/DNA/LNA gapmer with nine consecutive DNA nucleotides in the gap is a dose-dependent and sequence-specific inhibitor of the intercellular adhesion molecule-1 expression in the primary human umbilical vein endothelial cells [31]. In other experiments, LNA/DNA/LNA gapmers showed results as activators of RNase H-mediated RNA degradation and the known iso-sequential 2′-*O*-methyl gapmers [32].

#### 2.3.2. PNA

Peptide nucleic acid (PNA) was first synthesized by Danish scientists in 1991 [33]. PNA is a misnomer because it is not an acid. Unlike the phosphodiester backbone of DNA, RNA, and LNA, PNA has a peptide backbone usually built of N-(2-amino-ethyl)-glycine repeats. As a result, PNA possesses a unique combination of chemical, physical, and biological properties used in various antisense therapies. PNA can hybridize with RNA or DNA via Watson-Crick and also via Hoogsteen base pairing [34]. It is much more chemically stable and resistant to enzymatic decay than DNA and RNA. Due to the lack of electrostatic repulsion, PNA hybridizes with single-stranded RNAs and DNA molecules much more robustly and faster than the complementary DNA and RNA strands.

Moreover, PNA binds to nucleic acid target molecules both in vitro and in vivo. One PNA antisense nucleic compound, conjugated to the (RXR)_4_XB (cell-penetrating peptide), was targeted to *carA*, an essential gene for the MDR human pathogen *Acinetobacter baumannii*. The minimal medium in vitro experiments demonstrated that the PNA inhibited four strains at 1.25 μM concentration. The tested PNA compound did not affect the bacteria in lower concentrations [35].

#### 2.3.3. PMO

Morpholino oligonucleotides (PMOs) are neutrally charged DNA analogs with morpholino rings instead of ribose ones. Their syntheses are inexpensive and combined with characteristics such as solid nuclease resistance, binding affinity, and stability in serum and plasma, making PMOs a preferred object for pharmaceutical research. However, they have a lower melting temperature than DNA, therefore, higher concentrations are needed for successful inhibition. Also, their cellular uptake is inferior, but that can be significantly improved by attaching arginine-rich CPP to the PMO [36]. Other techniques to deliver PMO into the target cells are microinjection and scraping (electroporation or endosomal escape reagents). One designed and tested PMO reduces the neurofilament synthesis and inhibits axon regeneration in lamprey reticulospinal neurons [37]. Another two PMOs—peptide-conjugated PMO (PPMO) and non-conjugated PMO have been tested against Ebola virus infection in vitro and in vivo. The PPMO oligomers with a length of 22 nt targeted the translation start site region of EBOV VP35 positive-sense RNA and showed inhibitor effects in vitro. The in vivo experiments showed that PMO plus is an effective antisense oligonucleotide agent against Ebola infection in monkeys [38].

### 2.4. ASOs as Drugs

ASO-based technologies have certain advantages when applied to drug development for two main reasons. The mRNA is a universal target for drug development, which is present in every organism. Therefore, the antisense approach can be considered a versatile and universal drug development strategy. Also, we can easily design ASOs, which target predefined mRNA(s) using rational methods based on nucleic acid hybridization. Therefore, we can promptly engineer the ASOs that will down-regulate the expression of the desired gene.

Most medicines developed now are small molecules that specifically bind to a target molecule in the cell. Most of the target molecules for these drugs are proteins and some functional RNAs with complex 3D structures and form binding pockets where small molecules can specifically bind. Unfortunately, finding such small molecules that specifically bind their molecular targets in the cell is a time-consuming and costly process with no guarantee of success. Much more effort will be needed to find a small molecule that specifically binds a protein of interest and inhibits its function, a general and universal approach in drug discovery compared with the design of ASOs.

Half of the approved ASOs as therapeutic agents are splice-switching oligonucleotides (SSOs). They are short, modified synthetic antisense oligonucleotides complementary to pre-mRNA, disturbing the regular splicing repertoire of the transcript, causing a block of the RNA–RNA base-pairing or protein–RNA binding interactions [39]. The splicing of pre-mRNA is required to express most protein-coding genes, regulating gene expression and protein production. Splice-switching oligonucleotides target and alter the splicing in a therapeutic healing effect. In 2016, the U.S. Food and Drug Administration (FDA) approved another antisense drug called Eteplirsen (Exondys 51), a 30 nt neutrally charged PMO. Eteplirsen alters the splicing of the Duchenne muscular dystrophy pre-mRNA by hybridizing to exon 51 of the DMD gene, which leads to the correction of the translational reading frame and the production of shortened but functional dystrophin proteins. Duchenne muscular dystrophy is an X-linked recessive disease that affects one in 3500–5000 males, leading to progressive muscular deterioration that until now was untreatable [40]. Fortunately, with the approval of Eteplirsen, 14% of all DMD patients can be treated to slow down the progression of the disease. However, there has been controversy about the drug’s efficiency because of insufficient patients and inconsistent results [41,42]. That is why there are currently four additional trials to confirm its therapeutic effect: NCT01540409, NCT02255552, NCT02286947, and NCT02420379.

Three more nucleic acid-based drugs have been accepted and used to treat Duchenne muscular dystrophy. The first is Vyondys, also known as Dolodirsen injection, approved by the FDA in 2019. Vyondys also treats patients with DMD with a confirmed mutation in the dystrophin gene that can be treated by skipping exon. As a result, dystrophin production in skeletal muscle is increased. Viltepso, also known as Viltolarsen, is an approved therapy developed by NS Pharma, with its parent company Nippon Shinyaku (Kyoto, Japan), treating DMD resulting from mutations amenable to exon skipping. It is administered as an infusion into the bloodstream. Its application is possible at home as well as in the hospital. The FDA approved it in 2020. The third Amondys, known as Casimersen, was approved by FDA in 2021 as the first treatment for patients with DMD with a mutation amenable to skipping exon. That is the third Sarepta-approved RNA exon-skipping therapy for DMD after Exondys and Vyondys.

The FDA accepted one more ASO-based drug in December 2016 called Nusinersen (Spinraza). It is meant to treat spinal muscular atrophy (SMA), an autosomal recessive neurodegenerative disease affecting 1 in 10,000 live births and leading to motor neuron degeneration in the spinal cord and brainstem, concluding with muscle atrophy and general weakness [43,44]. Nusinersen is a second-generation modified ASO (2′-*O*-2-methoxyethyl phosphorothioate) administered mainly intrathecally into the CSF and modulates the splicing of SMN2 mRNA. This increases levels of the otherwise insufficient full-length SMN protein and helps patients with all three types of SMA. Moreover, it averts the manifestation of spinal muscular atrophy when treated earlier.

## 3. Synthetic Non-Coding RNAs as Therapeutic Agents

### 3.1. RNAi

**Figure 3:**
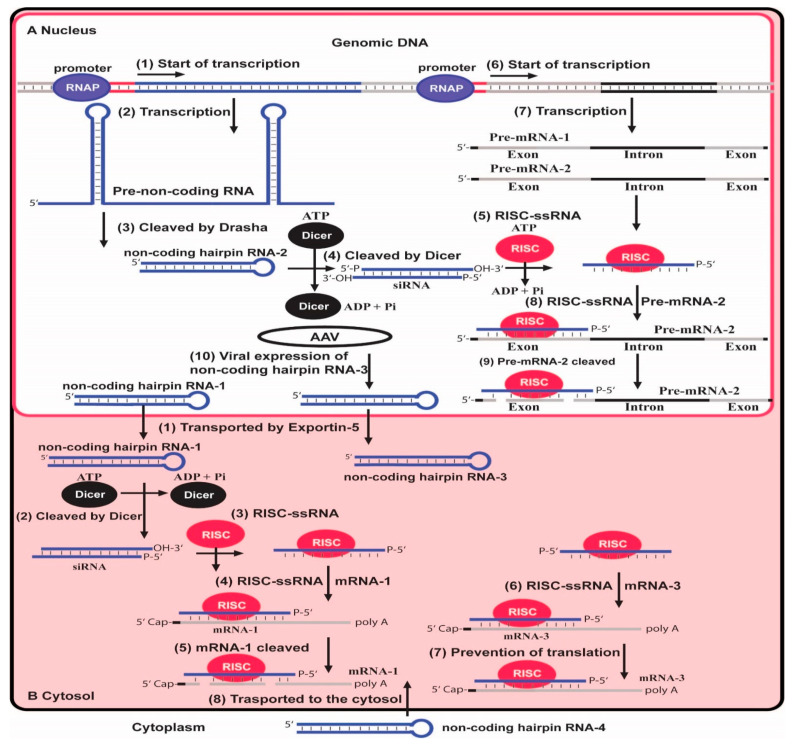
Control of gene expression in eukaryotes via the RNA interference pathway. (**A**) The genomic DNA in the nucleus has promoter sites responsible for pre-mRNA transcription. The binding of RNAP to the promoter sites triggers transcription (1) of DNA in pre-non-coding mRNA containing hairpin structures (2). Through the action of the Dicer enzyme, which is part of the RNA interference complex, the non-coding hairpin RNA-2 and hairpin RNA-1 are formed (3). The siRNA molecules are created by the action of the Dicer enzyme, which cleaves the dsRNAs into short dsRNA fragments—siRNAs (4). Due to the use of ATP by the Dicer enzyme, ADP and Pi are released in the reaction. siRNA is used as a template for recognizing complementary mRNA by RISC. The formation of the RISC-ssRNA complex requires ATP, so ADP and Pi are released (5). The binding of RNAP to the other promoter site (6) triggers the transcription of two pre-mRNAs, pre-mRNA-1 and pre-mRNA-2 (7). Pre-mRNA-2 is complementary to ssRNA from the RISC-ssRNA complex (8). By binding pre-mRNA-2, RISC is activated and induces cleavage. (**B**) The non-coding hairpin-RNA-1 produced from the Dicer enzyme and non-coding hairpin RNA-3 expressed from the viral vector AAV are transported from the nucleus into the cytosol via Exportin-5 (1). In the cytosol, the non-coding hairpin-RNA-1 is cleaved into siRNA molecules by the Dicer enzyme in an ATP-depended reaction (2). The formed RISC-ssRNA complex (3) binds to the complementary sequence mRNA-1 (4). The binding activates RISC and induces cleavage of mRNA-1 (5). RISC-ssRNA complex can also bind to non-coding mRNA-3 (6), which leads to the prevention of translation (7). RNA interference can be affected by incorporating a non-coding mRNA-4 from the cytoplasm inside the cytosol (8).

RNAi is found in a broad spectrum of eukaryotic organisms. Small interfering RNAs (siRNAs) are approximately 21–22 bp double-stranded molecules with two nucleotides overhanging at the 3′-end specific sequences and direct mRNA cleavage [45], which leads to inhibition of the translation of the targeted RNA due to its degradation. Therefore, synthetic siRNAs can be used in mammalian cells to tackle disease-causing genes (Figure 3). Synthetic non-coding RNAs can also specifically inhibit a target RNA via its microRNA (miRNA) function. For instance, short hairpin RNA (shRNA) and bifunctional short hairpin RNA (bi-shRNA) can inhibit target wild mRNAs or mutate mRNAs [46].

RNAi is also used to develop novel approaches for viral infections, cancer, and autoimmune diseases [47]. Approximately 20 clinical trials have been initiated using micro(mi)RNA and siRNA, and 19 ongoing trials are with siRNA only [48,49]. The first RNAi-based drug accepted and approved by the FDA (the U.S. Food and Drug Administration) is Patisiran (ONPATTRO™) [48,50,51,52]. Patisiran, a siRNA drug administered by IV infusion to treat hereditary TTR amyloidosis (hATTR), was accepted by the US FDA. hATTR is a medication for treating polyneuropathy caused by the rare lethal disease hereditary transthyretin-mediated amyloidosis. It is an effective gene-silencing drug. In the same month, another drug treating hTTR applied for NDA called Inotersen (ASO-based) by Ionis Pharmaceuticals (Carlsbad, CA, USA). As a result, the production of an abnormal form of transthyretin is inhibited (a genetic autosomal dominant disease caused by a mutation in the TTR gene, rapidly progressive, and affecting approximately 50,000 people worldwide). In November 2017, Alnylam Pharmaceuticals announced positive results from the APPOLO Phase 3 study and submitted an enrollment to the FDA for new drug discovery. In addition to their precise mechanism of action, siRNAs have higher specificity, higher potency, and greater reduced toxicity than protein-based drugs or other small molecules, making them apposite for cancer researchers [53,54].

Moreover, multiple genes (oncogenes, mutated tumor suppressors) can be targeted. More modifications are being constantly made to improve their biological safety, serum stability, off-target effects, and appropriate in vivo delivery [55]. For example, adding a 2′-modification of the ribose ring increases the endonuclease resistance of the siRNA.

Silenseed’s drug, siG12D-LODER, targets pancreatic cancer and, combined with chemotherapy, shows improvement in Phase I clinical trials [56]. In 2017, the company announced that they would continue with Phase II trials with clinical trials gov. identifier: NCT01676259. Another up-and-coming drug is RXI-109 by RXi Pharmaceuticals. It is a self-delivering siRNA that decreases the expression of connective tissue growth factor (CTGF) and improves the visual appearance of fibrosis and post-surgical scars. In August 2018, positive results were announced by Rxi Pharmaceuticals in Phase 1/2 clinical trial with RXI-109 for retinal scarring.

### 3.2. Guide RNA

CRISPR (clustered, regularly interspaced short palindromic repeats) is the name attributed to a family of DNA segments containing short repeated sequences from viruses, bacteriophages, or plasmids that have infected the bacterium in the past. CRISPRs are present in the locus CRISPR and other gene elements in bacteria and archaea. The bacterium uses short repeats to recognize and destroy the genomes of viruses similar to those that originated CRISPRs, thus constituting a form of acquired immunity system of prokaryotes [57]. CRISPRs are one of the essential elements of the CRISPR/Cas system, which is also involved in the acquired immunity of prokaryotes. The specificity of action of the CRISPR/Cas9 system is performed by a guide (g) RNA, which has specific primary and secondary structures.

A simplified version of this system has been created to provide a robust and precise genetic editing tool, which is much easier to use and, at the same time, cheaper than previous technologies. The new CRISPR-based strategies expand the possibilities, resulting in better diagnostics and environmental monitoring [58]. Thanks to the CRISPR/Cas9 system, it has been possible to modify the genes of multiple organisms permanently.

Recently, a technique was used to inhibit the urothelial cancer-associated 1 (UCA1) long non-protein-coding RNA by CRISPR/Cas9 to prove that the target has a role in the progression of bladder cancer [59]. UCA1 regulates embryonic development and bladder cancer invasion and advances as a regulator of the expression of different genes involved in tumorigenesis and embryonic development [60]. Several studies show that UCA1 is pivotal in anti-cancer drug resistance [61]. Its overexpression correlates with chemotherapeutic resistance (cisplatin, gemcitabine, EGFR-TKIs, imatinib, tamoxifen, and 5-FU). In that case, UCA1’s knockdown results in a drug sensitivity restoration and is expected to be a suitable diagnostic marker [61]. UCA1’s knockdown was revealed to restrain cell proliferation, migration, and invasion in large B-cell lymphoma by suppressing miR-331-3p expression [62]. It was also suggested as a possible medicinal target and biomarker for large B-cell lymphoma.

## 4. Functional Synthetic Nucleic Acids as Tools for Drug Discovery

### Synthetic Hammerhead Ribozymes as Therapeutics

**Figure 4:**
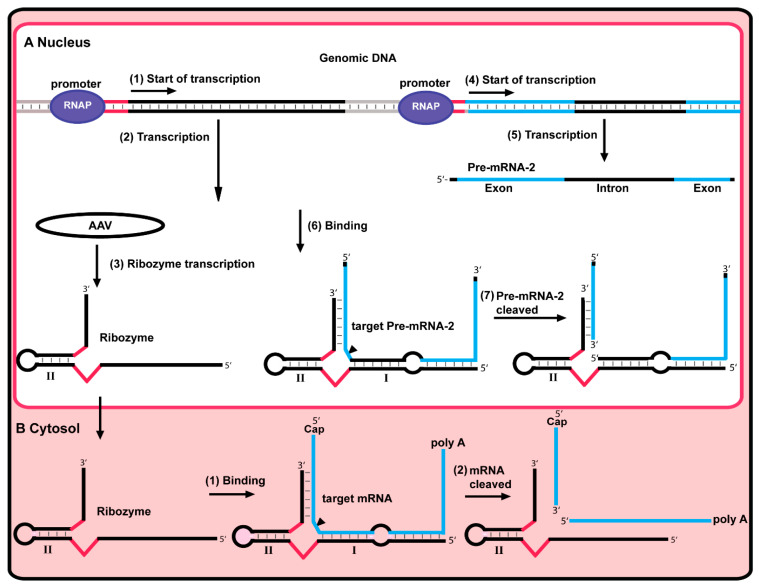
Control of gene expression by synthetic ribozymes. (**A**) Inside the nucleus, genomic DNA has promoter sites. The binding of RNAP to the first promoter site (1) triggers ribozyme transcription (2). A viral vector AAV in the nucleus (3) can also express the ribozyme. The binding of RNAP to the other promoter site triggers (4) the transcription of pre-mRNA-2 (5). The synthesized pre-mRNA-2 complement the ribozyme sequence and binds it (6). When bound to the pre-mRNA-2, the ribozyme is activated and cleaved at the cleavage site (black arrow). Along with that, the pre-mRNA-2 is also cleaved (7). (**B**) The ribozyme can also bind to the target mRNA inside the cytosol via complementary sequences (1). The ribozyme is activated and cleaves at the cleavage site (black arrow). Along with that, the mRNA is also cleaved (2), which leads to no translation of the mRNA.

Ribozymes are RNA enzymes that catalyze a chemical reaction like any other protein enzyme. They are highly applicable to manipulating various biological systems [63]. Since the identification of the first ribozyme in 1980 by Thomas R. Chech, applications of new synthetic ribozymes have been of great interest. The hammerhead ribozyme (HHRz) is one of the most frequently used types of catalytic RNA in drug discovery and development (Figure 4). Allosteric HHRz can be engineered as a biosensor by computational [64] and in vitro selection methods [65]. HHRz is a small ribozyme well-known for its capability of catalyzing the site-specific cleavage of a phosphodiester bond by hydrolyzes. HHRz consists of approximately 30 nucleotides that form 3 base-paired stems and a core of non-complementary nucleotides responsible for catalysis. The great interest in research in hammerhead ribozymes is due to their ability to block gene expression; therefore, they take place in developing new therapeutic agents [66].

Hammerhead ribozymes cleave the HIV-1 sequences in a cell-free environment in research. This has led to the development of human cells expressing the hammerhead ribozyme that, once treated with HIV-1, decreases the levels of HIV-1 gag RNA, which has been observed [67].

In gene therapy research, a HHRz was introduced to gastric carcinoma cells overexpressing a carrier protein responsible for multidrug resistance (MDR) in breast cancer cells (BPCR). The hammerhead ribozyme in question is an anti-BPCR agent that targets the BPCR-mRNA. By this method, the expression of the BPCR decreased dramatically (Figure 4) [68].

Investigations in trans-activating ribozymes showed that HHRz can be developed to attack the desired RNA and consequently block its gene expression. This represents a powerful tool in gene therapy against pathogens or genetic diseases [69]. There are results for the pseudoknot-type hammerhead ribozyme PK-HHRz, activated by a pseudoknot interaction between loops I and II, with higher cleavage activity than the wild-type sequence [70]. The increased activity of the pseudoknot-type hammerhead ribozyme PK-HHRz is achieved by elongating loop II. PK-HHRz could be used as a fundament for designing new variants of gene-regulating drugs. Recently, M1 ribozymes have been successfully used to target various RNA viruses in vitro and in vivo. This ribozyme is based on the RNA part of the RNase P of *E. coli* [71].

## 5. Factors Affecting Therapeutic Potency

Several essential factors generally affect the therapeutic potency of functional nucleic acids and ASOs, such as delivery in vivo, nuclease resistance, renal filtration, and toxicology.

### 5.1. Delivery

Although in the last 20 years, a lot of progress has been made in ASOs, one of the biggest challenges is still the successful delivery of the drug to its target in vivo [72]. The delivery of ASO in the human body depends on its generation. ASOs have different modifications and varying physical and chemical properties. Thus, they must be considered when choosing the methods for ASO delivery. Highly charged ASOs (PS-ASO) cannot passively diffuse across the lipid bilayer [23]. Examples of specific uptake of various charged and uncharged ASOs by different cultured cells and mouse models, known as gymnotic uptake, are exceptions to the rule. There are also results showing the uptake of uncomplexed ASOs, which occur in vivo in lung tissues [73].

There are different ways of administering ASOs, such as systematic applications that include subcutaneous, intradermal, intravenous, intrathecal, and topical applications. ASOs can be directly administered to cerebrospinal fluid (CSF) by intracerebroventricular or intrathecal infusion and via intranasal administration for delivery to brain cells. Once ASOs are in the organism, there are two main barriers to delivering them to their target. The first is the tissue barrier that the therapeutic ASO has to reach. Whether ASOs can successfully pass the vascular endothelial barrier, reticuloendothelial system (RES), blood-brain barrier, or renal excretion depends significantly on their size. Molecules transported between the blood and the parenchymal space are of limited size, up to 70 nm. Even if individual ASOs fit this criterion, they can still be removed by phagocytes or ultrafiltered by the kidneys. Different methods exist to avoid it, such as applying modified surfaces with polyethylene glycol or PS-ASOs that bind to specific plasma proteins [74]. The other main barrier is the permeation into the cells (cellular uptake), intracellular transport, and endosome escape. Generally, ASOs are taken into the cells by endocytosis, caveolar potocytosis, or pinocytosis.

There is an additional difference in the delivery of the ASO to its target cell depending on whether it is only the ASO or ASO attached to a carrier to facilitate the transportation. These carriers can be liposomes, lipids, nanoparticles, polymers, cell-penetrating peptides (CPPs), antibody conjugates, etc.

Liposomes and charged lipids are often used for ASO and siRNA delivery. Liposomes are spherical vesicles with at least one phospholipid bilayer [75]. On their inside, they are aqueous and can contain polar therapeutic molecules, including first and second-generation ASOs. On the other hand, cationic lipids are effective at transportation because they form strong complexes with ASOs due to their opposite charges. Liposomes are taken into the cells by membrane fusion, while the cationic lipids are via endocytosis.

CPPs have shown promising results as carriers of therapeutic molecules for different diseases. They are oligopeptides (6–30 residues) and can move through the cell membrane at low concentrations in vitro and in vivo [76]. They can be covalent or noncovalent to their cargo, but the covalent bond provides more stable conjugates [77]. It can be considered that negatively charged ASOs coupled with conjugated cationic CPPs could interact and lose their antisense or delivery properties. However, this does not affect neutrally charged ASOs such as LNA, PMO, and chimeric first and second-generation ASOs. Another occurrence is that the bond position matters, too, as CPP coupled at the 5′-end of the PMO is more active than at the 3′-end. CPP-PMO is an example of successful ASO transportation and general therapeutic effects, which passes the blood-brain barrier (BBB) and corrects aberrant splicing in ataxia-telangiectasia [78].

Antibodies have proven pharmaceutical significance as therapeutic agents. Specifically, monoclonal antibodies (mAbs) come from a single clone of cells designed to target specific antigens available in particular cells or tissues [79]. They can be used for conjugation with ASOs, and once administered, they are recognized by specific receptors, which will help them with cellular uptake by endocytosis. For instance, radioactively labeled anti-luciferase PNA conjuring to OX26 mAb successfully passes the blood-brain barrier and shows the luciferase-expressing brain tumors in rats in vivo [80].

N-acetylgalactosamine (GalNAc) is an amide derivative of the monosaccharide galactose. It involves intercellular communication and can be a targeting ligand of specific ASOs and siRNA. It binds to the asialoglycoprotein receptors on hepatocytes and ensures the introduction of the drug into the liver. Most of the recently approved siRNAs are conjugated with GalNAc. For example, GalNAC-siRNA conjugates have been used for delivery to the liver and eliminate the siRNA delivery problem for liver hepatocytes [81].

As many tissues can be reached only by systemic administration in vivo, the small RNAi therapeutic molecules must be conjugated with bigger carriers for filtration resistance and successful delivery to their target [82]. Such carriers are often non-viral vectors, such as nanotechnology-based ones. Among the frequently used are lipid-based—liposomes and lipoplexes. A cholesterol conjugate is another transporter that can carry inside the designed RNAi. As cholesterol is a component of the cell membranes, the conjugate passes quickly through the membrane and releases the RNAi inside the cell [83].

### 5.2. Stability

The drug’s half-life depends directly on its volume of distribution or on how widely it is distributed in the body. As much as the drug is widespread in the patient’s body, its half-life is longer. In addition, the half-life of this same drug is inversely dependent on its release from the body, which means the half-life is shorter when the drug release rate from the body is higher.

One of the most significant limitations in achieving the required effective dose of antisense oligonucleotide without reaching toxic therapy levels is that ASOs have a short cell half-life. That means the amount of drug needed to reach the effective dose will be achieved with a daily intake of common medications with a supporting role. Because the drug will be taken on a schedule, the half-life in the body plays a role to the extent that it determines the frequency of taking medicine. If we talk about drugs taken sporadically and at longer intervals than their half-life, the drug will not stay in the body long enough, and its short half-life will negatively affect its effectiveness.

DNA and RNA have phosphodiester backbones, which are susceptible to nuclease degradation. This limits the application of antisense oligonucleotides as therapeutic agents if they are not modified. The chemical modification of the ASO could enhance metabolic stability. The phosphorothioate modification (PS modification) is the most widely used modification, which replaces a single oxygen atom from the backbone with a sulfur atom. This chance increases the stability of the antisense oligonucleotides and enhances their binding affinity to their target [84]. Unmodified ASOs have a short half-life in vivo—around one hour in human serum. First-generation ASOs have half-lives of 9–10 h in human serum and 19 h in the cerebrospinal fluid of rats due to their PS modification and ability to attach to plasma proteins (thus, be safe from filtration). The LNA antisense oligonucleotide lifespan depends on the design of the chemical structure and could be up to 5–8 days. Laboratory tests showed that unmodified oligodeoxynucleotides had a half-life of 1.5 h. Another four LNA have been tested, increasing human serum lifespan ∼10-fold to ∼15 h. The main conclusion from the monitored results is that the stability of chimeric LNA/DNA oligonucleotides is much higher compared with 2′-*O*-methyl and phosphorothioate gapmers with half-lives of 12 and 10 h, respectively [32]. The half-life of the PMO-based Eteplirsen is 2 to 6 h in plasma.

Regarding RNA interference, unmodified siRNA has a shorter half-live in serum than the modified [83]. For example, it is proven that cholesterol conjugates added to siRNA increase their half-life in human serum and protect it from renal clearance. There are statistical analyses of PS-ASO experiments with enhanced antisense efficiency, which found motifs like: “CCAC”, “TCCC”, “ACTC”, “GCCA”, and “CTCT”. When the PS-ASO shows diminished antisense efficiency, found in motifs like “GGGG”, “ACTG”, “AAA”, and “TAA” [85].

### 5.3. Toxicity of ASOs

In contrast to many synthetic proteins, ASOs rarely induce or induce an immune response in humans, which is a significant advantage for their clinical usage. ASOs tend to produce transient toxicities in rodents, primates, and humans. Sometimes, however, the toxicities can have mild to moderate effects. The toxicities of ASOs can be sequence-independent or sequence-dependent. The sequence-independent toxicity is caused by backbone chemical modifications that may lead to unwanted non-specific protein binding. For instance, PS-ASOs may form disulfide bonds with peptides. A vigorous bioinformatics search can avoid the sequence-independent toxicities that can reduce off-target hybridization to a minimum during the designer stage of ASOs. The most common types of acute toxicity are high serum transaminase levels, partial thromboplastin prolongation, and transient activation of complement cascades. ASO toxicity can lead mainly to proinflammation, nephrotoxicity, hepatotoxicity unrelated to lysosomal accumulation, and thrombocytopenia [86]. There is a clear dependency between the accuracy and the thermodynamic stability of ASO/mRNA hybridization and ASO’s cytotoxicity published by us this year in Antibiotics [65]. These findings can be used by the design of the ASOs to reduce their non-specific cytotoxicity.

For example, the CpG-motif ASOs trigger a proinflammatory response, causing activation of Toll-like receptor 9, also known as TLR9. CpG motif ASO’s properties have been tested for therapeutics for cancer therapies. However, antisense oligonucleotides have a specific design and minimize the proinflammatory responses by avoiding CpG motifs. They can elicit a proinflammatory reaction if their dose level is high in the body and weaker than the CpG-motifs [87].

The human immune system possesses a specific innate immune pathway that senses cytosolic DNA [88]. It is known as the STING pathway and is responsible for activating downstream signaling events such as interferon regulatory factor 3 (IRF3) activation and human interferon-beta protein (IFN-β) gene expression [88]. IFR3 is an analog of the interferon regulatory factors 1 and 2, with functional domains like a nuclear export signal, a DNA-binding domain, a C-terminal IRF association domain, and regulatory phosphorylation sites. It is part of the interferon transcription factors family. In the cytoplasm, it is inactive. After phosphorylation with serine or threonine, it forms a complex, which translocates to the nucleus. It manifests its transcriptional activity role affecting the interferons alpha and beta genes [89]. The fibroblasts‘ antiviral activity mainly involves the innate immune response by producing IFN-β proteins. Multiple post-translational modifications regulate different steps of the STING pathway. The STING pathway detects sequence-nonspecific cytosolic DNA species with more than ~70 bp in human cells [90]. It also senses different RNAs, cyclic-di-GMP, and cyclic-di-AMP generated by numerous intracellular bacteria, such as *Listeria monocytogenes*, essential in microbial pathogenesis mechanisms of host defense causes of inflammatory disease and cancer.

## 6. Riboswitches as a Target for Antibacterial Drug Discovery

Riboswitches are strongly conservative gene control elements, primarily found in the 5′-UTR region of mRNA, where they control the gene expression of some vitamin precursors like riboflavin, thiamin, and cobalamin, amino acids like methionine and lysine, the synthesis of some nucleotides like adenine, and guanine, and other essential metabolites by two main regulatory mechanisms such as termination of transcription, and prevention of translation [91,92]. Some riboswitches regulate gene expression by trans-acting regulatory mechanisms and self-cleavage. As biosensors, they sense the presence of small molecules and bind to specific essential ligands that trigger conformational changes or have an essential role in biofilm formation [93]. The riboswitches are found in many bacteria, archaea, plants, and fungi but are still not in the human genome. There are criteria to classify the suitability of each riboswitch class for targeting antibacterial drugs [10,94]. The first one is the riboswitch found in human bacterial pathogen bacteria. The second is the riboswitch to control the biochemical pathway(s) to synthesize essential metabolites in the bacterium, which does not have an alternative biosynthetic pathway without riboswitch control.

The third criterion is the synthesis of transporter protein for the essential metabolite to be under riboswitch control. It must fulfill all requirements to be classified as a promising riboswitch class for an antibacterial target. The design of therapeutic molecules, such as ASOs, which can bind in vivo to the aptamer domain, might be the answer to successfully creating entirely new classes of antibiotics [64]. Recent studies prove the inhibition effect of chimeric ASOs on the bacterial growth of *S. aureus* targeting SAM-I riboswitch [95]. The combined application of the first two ASOs, which target the glucosamine-6-phosphate (*glmS*) riboswitch and the *nagA* mRNA, block the synthesis of glucosamine-6-phosphate entirely and inhibits the bacterial growth of *S. aureus* [3]. Other engineered ASOs have been tested as antibacterial agents that target the flavine mononucleotide (FMN) riboswitch and inhibit the growth of *E. coli*, *L. monocytogenes*, and *S. aureus* [96]. Moreover, we have successfully targeted thiamine pyrophosphate (TPP) riboswitch in *S. aureus* for antibacterial drug development [97]. The compounds with proven antibiotic effects include Roseoflavin (RoF) and 8-dimethyl-8-amino-riboflavin (AF), where the latter has lower toxicity [98].

## 7. Prospective of Applying Antisense Nucleic Acid-Based Strategies for Drug Development

Antisense nucleic acid-based therapies can offer suitable treatment for genetic disorders or infectious diseases. The antisense therapies must be adaptable, precisely created, and selectively target the specific gene(s). ASOs are stable single-stranded molecules that directly bind to the targeted mRNA by penetrating different tissues and cells when modified and attached to a cell-penetrating protein. When an RNA(s) sequence is known to be causative of a specific disease, it is possible to prevent the function of this RNA(s) by introducing different types of antisense nucleic acids in the cell. Many different types of nucleic acids, such as PS-DNA, LNA, PNA, and other modified DNA oligomers, can be employed in various antisense therapeutic strategies. These methods are generally based on Watson-Crick’s complementary base-pairing between the ASOs and the targeted mRNA. We can promptly design and synthesize such antisense oligonucleotides. Furthermore, well-established methods exist for antisense oligonucleotide delivery in various cell types.

Moreover, we use various bioinformatics databases to find the most suitable (specific) part of the targeted mRNA. Such databases include KEGG for biochemical pathways, GeneBank for DNA and RNA sequences, Rfam, and Rswitch [99]. This will allow us to achieve a high selective antisense oligonucleotide-based inhibition, which can avoid non-specifically targeting other RNAs and, therefore, may reduce many adverse effects of our particular ASOs. Thus, we apply rational approaches to select our RNA targets [10].

In addition, we have established computational algorithms [11] and software [100] for the design of allosteric ribozymes and postulated rational rules for the design of ASOs that have all been proven to be over 90% successful [64]. They are all based on computing secondary RNA structures and DNA/RNA hybridization using the partition function for RNA folding in conjunction with thermodynamic parameters.

Since synthesizing the first ASOs in the 1960s, a few drugs based on antisense technology have been approved for patient treatment. The first approved drug is Fomivirsen (Vitravene), a 21 nt-long PS-modified oligonucleotide. Its role was local cytomegalovirus (CMV) retinitis treatment in patients with acquired immunodeficiency syndrome (AIDS) by targeting CMV mRNA and inhibiting essential viral proteins [101]. However, in 2002, it was withdrawn from the European Union market for commercial reasons [102].

The second drug is mipomersen (Kynamro), a second-generation 20 nt-long chimeric ASO approved in 2013 and accu{ulated in the liver (Table 1) [103,104]. It inhibits apolipoprotein B-100 and increases the survival of patients with a rare genetic disease called homozygous familial hypercholesterolemia (HoFH). HoFH is an autosomal dominant disease that leads to increased low-density lipoprotein (LDL) and higher risks for atherosclerosis and cardiovascular disease in approximately 1/1,000,000 people in Western Europe [105]. While it has been approved only for the homozygous form of the disease, it is also being tested for its therapeutic role in the heterozygous form of HoFH.

A new antisense drug already applied to a rolling submission to the FDA is alicaforsen to treat pouchitis. It is a 20-base oligonucleotide that targets the intracellular adhesion molecule-1 (ICAM-1) mRNA. It shows promising results in patients with pouchitis and other inflammatory diseases, such as left-sided ulcerative colitis or proctitis.

Many antisense oligonucleotides are being designed and tested for their therapeutic effects in cancer, neurodegenerative diseases, and cardiovascular or metabolic diseases. As of this moment, one of the biggest pharmaceutical companies, Ionis (previously known as ISIS), has over a thousand patents on RNAi and antisense oligonucleotides and 47 ASO drugs (according to the Ionis Pharmaceutical pipeline website) under different phases of clinical trials, including Volanesorsen, Inotersen (applied for NDA in November 2017) and IONIS-HTTRx. The ASOs are in the cardio-renal, metabolic, neurological, infectious diseases, rare cancer, ophthalmology, pulmonary and allergy, hematology, and other therapeutic areas. Currently, there are 16 FDA-approved drugs based on oligonucleotides. Ten are based on ASOs, with two withdrawn (Table 1), 5 are based on siRNAs, and 1 is based on an aptamer and is withdrawn. ASO-based drugs have the highest number among all oligonucleotide-based FDA-approved drugs. The main reason for that is the single-stranded nature of ASO in contrast to siRNA and the lack of secondary and tertiary structures in contrast to the aptamers.

Regarding antibacterial resistance and overgrowing global problems, antisense oligonucleotides inhibit the expression of crucial genes in pathogenic bacteria, leading to their death [106]. Examples of such ASOs are third-generation PNA and PMO, which, combined with CPP, have a much increased cellular effect in vitro and in vivo [8,107]. Therefore, there are reasonable expectations that some ongoing preclinical experiments and clinical trials will successfully produce approved medicines.

In recent years, artificial intelligence (AI) has had an increasing number of applications in drug discovery. Various techniques, such as reasoning, solution search, and machine learning (ML), are parts of AI, and ML applies algorithms that recognize patterns in a database. An essential area of ML is deep learning (DL) based on artificial neural networks (ANNs) [108]. AI algorithms such as solution search and ML can establish promising RNA targets in different diseases using ASOs or ribozymes. ML is applied for drug design and target discovery [109].

Moreover, such AI algorithms can be employed for side effects and toxicity prediction of ASOs, which are significant problems in light of the broad applications of ASOs as therapeutic agents. In addition, HTS and HCS arrays can be used to test side effects and the general and specific toxicity of many ASOs [110]. Such arrays are also applicable for fully automated evaluation of the efficiency of various methods for ASO delivery, general toxicity, and specific RNA inhibition in various cells.

Several issues are using ASOs to develop new drugs, including the half-life of ASOs in vivo, toxicity, accuracy, and delivery methods that can be tackled rationally. For instance, the half-life of ASOs in vivo can be increased by increasing their molecular weight by reducing the renal filtration rate of ASOs. The main advantage of ASO technology is that all these issues can be tackled rationally and systemically because the primary mechanism of action of ASOs is based on DNA/RNA hybridization with Watson–Krick hydrogen bonding that is easy to predict and engineer. The ASOs can quickly be delivered in the cell if coupled with CPP [3,97].

The AI and genome-wide analyses can be used to find and avoid the mis-hybridization of ASOs to unintended RNAs. To address this problem, thermodynamic and kinetic parameters can be computed [11] for the gap between the perfectly matching ASO and its RNA target and the unintended RNA(s). In addition, the non-specific binding of some thiol-modified ASOs from the first generation to proteins can be limited by reducing the number of thiol-modified nucleotides or applying second or third generations of ASOs.

Kidney filtration removes the ASOs from the bloodstream within several hours, which worsens their pharmacokinetic properties to increase the molecular weight of the ASOs and decrease the rate of their kidney filtration; glycoproteins can be attached to them. There are many different glycoprotein types; some aggregate with other proteins, causing problems. Therefore, the glycoproteins used for attachment to ASOs must be chosen wisely, considering the possibility of binding to other proteins in the cell. It is also possible to use proteins that target a specific cell type. Apart from that, there are three other theoretical possibilities for the cell to develop resistance against the ASO that are very difficult to make because more than one mutation in many genes is needed for the cell to degrade the ASO quickly to export it outside the cell, etc.

To achieve significant inhibition, the type of ASO must be carefully chosen following the targeted RNA expression level. For instance, if the targeted RNA has a relatively low expression level, a single-turnover acting ASOs of the second or third generation can be employed. On the contrary, if the targeted RNA has a relatively high expression level, multi-turnover acting ASOs via RNase H of first or chimeric of first and second-generation can be applied. Moreover, such ASOs can also work via RNAse P if three nucleotides CCA at the 3′-terminus of ASOs do not hybridize with the targeted RNA. In addition, the ASOs must not self-hybridize and form stable secondary structures to achieve high inhibition efficiency because that will prevent them from hybridizing with the targeted RNA.

The targeted site of the RNA has to be single-stranded to be fully accessible for hybridization with the ASO. This can be assessed with programs for the computation of RNA secondary structures to reduce the possibility of mutations in the targeted RNA site that are non-complementary to the ASO, thus the targeted RNA sequence must be highly conserved. However, mutations in the targeted RNA can arise, rendering the ASO cab inefficient. When this happens, the targeted RNA can be sequenced, and the ASO sequence can be altered to complement its targeted RNA site.

Administration and delivery methods are essential in applying ASOs in vivo [111]. ASOs can be used locally via topical administration or intramuscular or intravenous injections. ASOs can enter the cell via attached cell-penetrating oligopeptides (CPPs) and various nanoparticles. Some of the carriers can have specificity to particular cells. For instance, CPPs enter only bacterial cells, reducing the side effects of antibacterial ASOs on human cells. There are 5 siRNA-based drugs and only 1 aptamer-based drug, which is withdrawn (Table 2).

## 8. Conclusions

Functional and antisense nucleic acids are essential molecules for drug discovery and development. They can be used as drug targets and discovery and development tools. The results from the Human Genome Project showed that only ∼2% of the human genome encodes for proteins; the rest are noncoding RNAs. Many efficient methods exist for delivering nucleic acid oligomers in vitro and in vivo. Modern nucleic acid chemistry lets us synthesize various oligomers, inducing modified DNAs and RNAs, LNAs, and PNAs [112]. These four oligonucleotides possess different thermodynamic stabilities when hybridizing to target RNA. They have other methods for administration, half-life time in the cell, and pharmacokinetic properties. Therefore, antisense nucleic acid technologies offer flexible tools that can successfully adapt to a broad range of clinical trials. These nucleic acids can be used as ASOs based on Watson-Crick’s complementary base pairing to target any mRNAs in the cell.

Since mRNAs are present in all life forms, antisense technologies can be regarded as versatile tools for drug development. The full potential of these technologies will be reached in the next several years since there are over 20 antisense drug candidates in various phases of clinical trials. These clinical trials tackle various disorders and include antiviral and anti-cancer treatments. Several ASO drugs, such as Mipomersen, Eteplirsen, Vyondys, Viltepso, and Amondys, are in use, and their number is expected to grow. Functional nucleic acids such as riboswitches and ribozymes serve as molecular targets and tools for drug development. At least 28 riboswitches regulate the gene expression of many critical biochemical pathways in 59 human bacterial pathogens [1,113]. Rational designer methods can be employed for all functional and antisense nucleic acids for drug development with a 100% success rate [64] that can benefit the pharmaceutical industry.

## Figures and Tables

**Table 1:** ASO-based drugs approved by the FDA. There are 10 ASO-based drugs approved, of which the first 2 are withdrawn.

No.	Names	Target	Year of Approval	Administration	Chemical Modification	Company
1	Fomivirsen (Vitravene^®^)	Cytomegalovirus—the gene for CMV immediate-early 2 protein	1998 Withdrawn	Intravitreal	PS	Ionis (Carlsbad, CA, USA)
2	Mipomersen (Kynamro^®^)	Hypercholesterolemia (FH)—the gene APOB encoding apolipoprotein B	2016 Withdrawn	Subcutaneous	2′-O-MOE, PS	Genzyme (Cambridge, MA, USA)
3	Eteplirsen (Exondys 51^®^)	Duchenne muscular dystrophy (DMD)—Rescue the expression of dystrophin through exon-51	2016	Intravenous	PMO	Sarepta (Cambridge, MA, USA)
4	Nusinersen	exon-7 inclusion of the mRNA of SMN2 gene	2016	Intrathecal	2′-O-MOE, PS, 5-methyl cytosine	Biogen (Cambridge, MA, USA)
5	Inotersen (Tegsedi^®^)	Hereditary transthyretin (TTR) amyloidosis	2018	Subcutaneous	2′-O-MOE, PS	Ionis (Carlsbad, CA, USA)
6	Milasen	DMD—dystrophin through exon-45	2018	Intrathecal	2′-O-MOE, PS, 5-methyl cytosine	Boston Children’s Hospital (Cambridge, MA, USA)
7	Golodirsen (Vyondys 53^®^)	DMD—rescue the expression of dystrophin through exon-53 of DMD gene	2019	Intravenous	PMO	Sarepta (Cambridge, MA, USA)
8	Waylira (Volanesorsen)	Apolipoprotein C3	2019	Intravenous	2′-O-MOE	Akcea Therapeutics (Cambridge, MA, USA)
9	Viltolarsen (Viltepso)	Exon 53 of DMD	2020	Intravenous	PMO	NS Pharma (Kyoto, Japan)
10	Casimersen (Amondys 45)	Exon 53 of DMD	2021	Intravenous	PMO	Sarepta (Cambridge, MA, USA)

**Table 2:** siRNA and aptamer-based drugs approved by the FDA. There are 5 siRNA-based approved drugs and only 1 aptamer-based drug, which is withdrawn.

Type	Drug	FDA Approval	Company	Disease
siRNA	Patisiran	2018	Ionis (Carlsbad, CA, USA)	Hereditary transthyretin-mediated amyloidosis
	Givosiran	2019	Alnylam (Carlsbad, CA, USA)	Acute hepatic porphyria
	Lumasiran	2020	Alnylam (Carlsbad, CA, USA)	Primary hyperoxaluria type 1
	Inclisiran	2021	Novartis (Basel. Switzerland)	Primary hypercholesterolemia
	Vutrisiran	2022	Alnylam (Carlsbad, CA, USA)	Hereditary transthyretin-mediated amyloidosis
Aptamer	Pegaptanib	2004 (withdrawn)	Pfizer/Eyetech (New York City, NY, USA)	Neovascular (wet) age-related macular degeneration
